# Structural Insights into the Ligand-Binding and -Releasing Mechanism of *Helicoverpa armigera* Pheromone-Binding Protein PBP1

**DOI:** 10.3390/ijms23031190

**Published:** 2022-01-21

**Authors:** Jiangge Zheng, Meiting Yang, Kun Dong, Jianbo Zhang, Huali Wang, Mengjia Xie, Wei Wu, Yong-Jun Zhang, Zhongzhou Chen

**Affiliations:** 1 State Key Laboratory of Agrobiotechnology, College of Biological Sciences, China Agricultural University, Beijing 100193, China; zhengjiangge@cfsa.net.cn (J.Z.); zb20163020118@cau.edu.cn (M.Y.); sz20203020189@cau.edu.cn (M.X.); wuweiyou@cau.edu.cn (W.W.); 2 China National Center for Food Safety Risk Assessment, Beijing 100022, China; jianbozhang@cfsa.net.cn (J.Z.); wanghuali@cfsa.net.cn (H.W.); 3 State Key Laboratory for Biology of Plant Diseases and Insect Pests, Institute of Plant Protection, Chinese Academy of Agricultural Sciences, Beijing 100193, China; dongkun031@163.com (K.D.); yjzhang@ippcaas.cn (Y.-J.Z.)

**Keywords:** *Helicoverpa armigera*, pheromone-binding protein, crystal structure, ligand binding and releasing mechanism, complex, acidic pH, (Z)-9-hexadecenal, pheromones, mechanistic insights, fluorescence binding assays

## Abstract

Cotton bollworm (*Helicoverpa armigera*) is a worldwide agricultural pest in which the transport of pheromones is indispensable and perceived by pheromone-binding proteins (PBPs). However, three-dimensional structure, pheromone binding, and releasing mechanisms of PBPs are not completely illustrated. Here, we solved three structures of the cotton bollworm HarmPBP1 at different pH values and its complex with ligand, *Z*-9-hexadecenal. Although apo-HarmPBP1 adopts a common PBP scaffold of six α-helices surrounding a predominantly hydrophobic central pocket, the conformation is greatly distinct from other apo-PBPs. The *Z*-9-hexadecenal is bound mainly by hydrophobic interaction. The pheromone can enter this cavity through an opening between the helices α5 and α6, as well as the loop between α3 and α4. Structural analysis suggests that ligand entry into the pocket is followed by a shift of Lys94 and Lys138, which may act as a lid at the opening of the pocket. Acidic pH will cause a subtle structural change of the lid, which in turn affects its ligand-binding ability, differently from other family proteins. Taken together, this study provides structural bases for the interactions between pheromones and PBPs, the pH-induced conformational switch, and the design of small inhibitors to control cotton bollworms by disrupting male–female chemosensory communication.

## 1. Introduction

Cotton bollworm (*Helicoverpa armigera*), an important agricultural pest, mainly damages crops such as cotton, corn, peanuts, and soybeans and seriously affects the planting and production of crops. It is currently a serious problem as to how to continuously and effectively reduce the loss caused by cotton bollworms. Controlling this insect pest may be achieved by interfering with olfactory pathways to block detection of female-produced sex pheromones and thus disrupt mating [1].

Insects have evolved a sensitive olfactory system to detect information-rich odor molecules for their survival and reproduction [1]. The initial step of odorant recognition involves binding odorant molecules to the odorant binding proteins (OBPs) and carrying them to odorant receptors (ORs). These OBPs may serve as molecular targets for attracting moths or other insect species [1,2]. Lepidoptera OBPs have been divided into pheromone-binding proteins (PBPs) and general OBPs (GOBPs) [3].

Many insect PBPs structures have been solved both in the crystal forms and in solution since the first crystal structure of silkworm moth BmorPBP/bombykol complex was reported [4,5,6,7,8]. These structures exhibit many identical characteristics, including six or seven α-helices, three strictly conserved disulfide bridges, and a hydrophobic binding pocket. However, growing evidence suggested that these insect PBPs have significant differences in ligand binding and releasing mechanisms because of their different cavity shapes and openings. The NMR structure of silkworm moth BmorPBP proved that the C terminal dodecapeptide segment of the acidic BmorPBP structure (pH 4.5) formed an additional α-helix in the protein core, occupying the corresponding pheromone-binding site and extruding ligands [5,9] (Figure S1A). The study suggested that the C-terminal region plays a key role by forming a helical structure to replace the corresponding pheromone-binding pocket at low pH. Moreover, at neutral pH, the additional helix withdraws from the binding pocket and favors pheromone binding [2]. pH-triggered conformational switch involving histidine(s) protonation/deprotonation is a regulatory mechanism [10]. The potential importance of the histidine residues for PBP function was first suggested in *B. mori* on the basis of histidine positions in the crystal structure [5]. Interestingly, His69, His70, and His95 are identical in lepidopteran PBPs and GOBPs, suggesting that pH-triggered conformational switch may be conserved for the entire order.

The interactions between ligands and insect OBPs have also been proposed. The structure of BmorPBP/bombykol complex revealed that Ser56 specifically interacts with the ligand in the binding pocket [5]. Other research on *A. polyphemus* PBP indicates that Trp37 may play an important role in the initial interaction with the ligand, while Asn53 plays a critical role in the specific recognition of pheromones [10,11].

In *H. armigera,* three PBPs were identified, and their abilities to bind five pheromone components were measured by fluorescence-binding assay [12]. It was shown that HarmPBP1 binds the two principal pheromone components with strong affinities. However, there was no three-dimensional structural information reported on HarmPBPs. To better understand the pheromone–HarmPBP1 binding mechanism and to design a stable synthetic sex pheromone useful as a mating disruptor, we determined crystal structures of HarmPBP1 at different pH values and in complex with the main constituent of the sex pheromone *Z*-9-hexadecenal (*Z*9-16:Ald). Structural analysis of the complex revealed the important residues of HarmPBP1 in binding *Z*9-16:Ald. These key residues were consistent with the site-directed mutagenesis experiments and fluorescence binding assays. These results reveal a novel mechanism for ligand release at acidic pH.

## 2. Results

### 2.1. Crystal Structures of Apo-HarmPBP1 and HarmPBP1/Z9-16:Ald Complex

**Figure 1:**
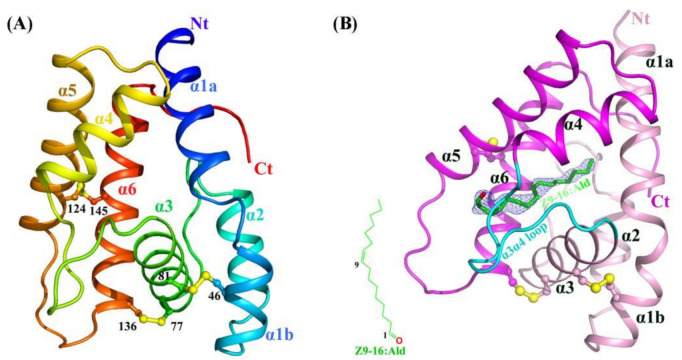
Crystal structures of apo and *Z*9-16:Ald-bound HarmPBP1. (**A**) Ribbon representation of apo-HarmPBP1 with rainbow coloring mode. (**B**) The *Z*9-16:Ald-bound HarmPBP1 structure. The chemical structure of *Z*9-16:Ald (green) is shown in the bottom left. The differential electron density for the *Z*9-16:Ald in a Fo-Fc map is contoured at 2.5 σ (light blue). Disulfide bridges, yellow; α3α4 loop, cyan; Nt: N-terminus, Ct: C-terminus.

HarmPBP1 was successfully expressed in bacteria and then purified through Ni-affinity chromatography, ion exchange chromatography, and size-exclusion chromatography (see the Methods and Materials). The highly purified protein (Figure S2A) produced small but regular crystals in crystallization trials. After optimizing crystallization conditions, we succeeded in obtaining crystals. The apo-HarmPBP1 structure at pH 7.5 was determined by molecular replacement and refined to a resolution of 1.3 Å in the space group of *P*2_1_, with an *R_work_* of 17.9% and an *R_free_* of 19.8% (Table 1). The cloned protein was 144 residues long and possessed six cysteine residues. The structure was built in the electron density map, except for the C-terminal residues 159-170. The HarmPBP1 scaffold contains three conserved disulfide bonds linking α-helices α1 and α3 (Cys46-Cys81), α3 and α6 (Cys77-Cys136), and α5 and α6 (Cys124-Cys145) (Figure 1A), encapsulating the hydrophobic pocket for pheromones binding.

On the basis of the affinity data of HarmPBP1 to different pheromone components [12], we chose *Z*9-16:Ald and *Z*11-16:Ald with the strongest affinity for complex crystallization trials. Through a series of cocrystallization experiments, we eventually obtained cocrystals of HarmPBP1 with *Z*9-16:Ald,   probably because *Z*9-16:Ald bound slightly stronger than *Z*11-16:Ald. HarmPBP1/Z9-16:Ald complex crystal had different morphologies from that of the apo-HarmPBP1, indicating the formation of the complex with ligand *Z*9-16:Ald. We collected a dataset at 2.09 Å resolution. The binding of *Z*9-16:Ald was confirmed by the differential electron density (Figure 1B), and the crystal packing between the apo at pH 7.5 and the complex was different. The overall structure of the complex was similar to the apo-HarmPBP1, and the root-mean-square deviation (RMSD) between these two structures was as low as 0.4 Å. Structural variation was only observed in a few loop regions (Figure S2B), showing that they were almost identical. The most prominent structural changes were the loop between α3 and α4 (α3α4 loop), with a 0.7 Å shift and the loop between α5 and α6 (α5α6 loop) with a 1.9 Å shift. Moreover, an additional three residues were solved in the C-terminus.

### 2.2. Structural Comparison Revealed a Specific Conformation of Apo-HarmPBP1

**Figure 2:**
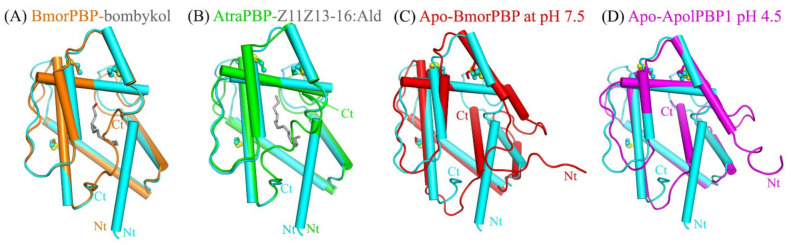
Superimposition of apo-HarmPBP1 (cyan) on selected OBPs. (**A**) BmorPBP-bombykol (PDB: 1DQE, orange). (**B**) AtraPBP-*Z*11*Z*13-16:Ald (PDB: 4INW, green). (**C**) apo-BmorPBP at pH 7.5 (PDB: 2FJY, red). (**D**) Apo-ApolPBP1 at pH 4.5 (PDB: 2JPO, magenta). Conserved disulfide bonds (yellow) are shown in ball-stick modes. Ligands, gray.

A Dali server search of the apo-HarmPBP1 structure identified BmorPBP-bombykol (PDB 1DQE, *Z* = 23.5, RMSD = 1.0 Å) and AtraPBP1-*Z*11*Z*13-16:Ald (PDB 4INW, *Z* = 22.4, RMSD = 1.2 Å) [5,13] as the closest structural homologs (Figure 2 ). Surprisingly, the structural similarity between apo-HarmPBP1 and all other apo-GOBPs or apo-PBPs was lower as the RMSD was larger than 1.5 Å, revealing a distinct conformation of apo-HarmPBP1 compared with other apo-PBPs.

The secondary structures of HarmPBP1 are quite similar to those of BmorPBP in *Bombyx mori* [5,9,14], ApolPBP1 in *Antheraea polyphemus* [10]*,* and AtraPBP1 in *Amyelois transitella* [13] (Figure S3). The most significant structural differences between apo-HarmPBP1 and other known insect PBPs were observed at the C terminus. The last 12 residues were missed in the structure of apo-HarmPBP1, which might have been a result of crystal packing. Both BmorPBP-bombykol (PDB ID:1DQE) and AtraPBP1-*Z*11*Z*13-16:Ald (PDB ID: 4INW) had a C-terminal loop [5,13], while a C-terminal helix was found in the apo-BmorPBP at pH 7.5 (PDB ID: 2FJY) and ApolPBP1 at pH 4.5 (PDB ID: 2JPO) [9,10] (Figure 2). Then, we compared HarmPBP1 with these two types of PBPs mentioned above. The high structural similarity between HarmPBP1/*Z*9-16:Ald complex and BmorPBP-bombykol or AtraPBP1-*Z*11*Z*13-16:Ald complex (Figure 2) suggests that they may share a similar ligand-binding mode. However, the conformations of both termini as well as some loops differed significantly between apo-HarmPBP1 and the apo-BmorPBP/apo-ApolPBP1 (Figure 2C,D). The first helix of HarmPBP1 occupied the C-terminal helix position of the above two structures. Moreover, the seventh helix of the latter was located in the protein core. Finally, for BmorPBP and AtraPBP1, the structural changes between the apo and its corresponding complex were evident (comparing Figure 2A with Figure 2C), but it was subtle for HarmPBP1 (Figure S2B).

### 2.3. The Binding of Ligand in the HarmPBP1 Binding Cavity

**Figure 3:**
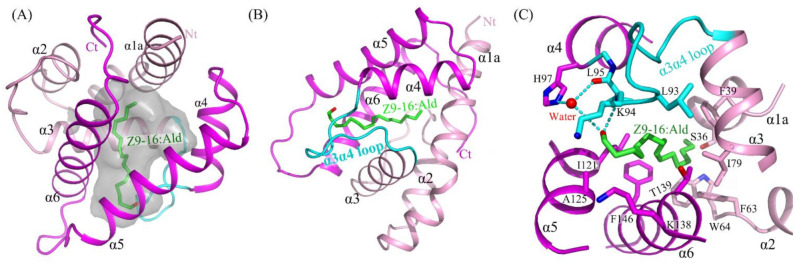
The binding pocket and its binding site details. (**A**) HarmPBP1 has a “C” shaped ligand-binding cavity. (**B**) The binding of *Z*9-16:Ald (green) in the HarmPBP1 binding pocket. (**C**) Detailed interaction of *Z*9-16:Ald. Hydrogen bonds, dashed lines. α3α4 loop, cyan.

The structure of HarmPBP1 was found to contain a hydrophobic cavity formed by the five helices α1, α3, α4, α5, and α6. The cavity volume of the HarmPBP1 complex was 206 Å^3^. The cavity had a “C”-shape (Figure 3A), and the ligand *Z*9-16:Ald fitted perfectly inside the cavity (Figure 3B). The HarmPBP1/*Z*9-16:Ald complex contained a single molecule of *Z*9-16:Ald that was stabilized primarily by an array of hydrophobic interactions, which were mediated by the side-chains of Phe63, Ile79, Leu93, Leu95, Ile121, and Phe146 (Figure 3C). In addition, a stacked arrangement of phenylalanines at positions 39 and 146 interacted with the ligand near the desaturated carbons (Figure 3B,C). The five aromatic residues Phe39, Phe63, Trp64, Phe103, and Phe146 were strictly conserved in all known lepidopteran PBPs (Figure S3). These residues and the shape of the cavity were therefore likely responsible for the specific binding of the unsaturated aliphatic odorant molecules. Moreover, Ser36 and Thr139 also interacted with *Z*9-16:Ald through van der Waals contacts.

**Figure 4:**
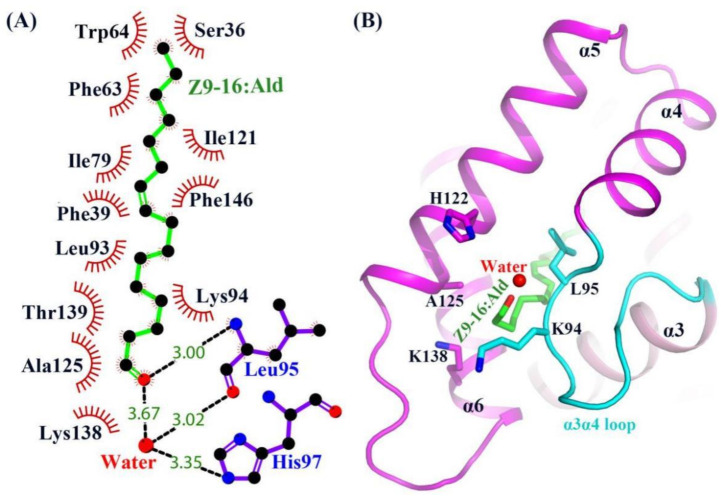
Structure of the *Z*9-16:Ald bound state of HarmPBP1. (**A**) LIGPLOT diagram [15] of *Z*9-16:Ald. Hydrogen bonds are represented by dashed lines and hydrophobic contacts by arcs with radiating spokes. Atoms involved in hydrophobic contacts are represented by black circles. (**B**) Ribbon diagram of HarmPBP1. A single *Z*9-16:Ald molecule (green) binds in the central cavity and enters through an opening formed by α5, α6, and the α3α4 loop.

Except for the main hydrophobic interaction, hydrogen bonds were also observed in the HarmPBP1/*Z*9-16:Ald complex. The oxygen atom of the ligand aldehyde group formed a hydrogen bond with the main chain of Leu95. The aldehyde group also bound Leu95 and His97 through weak water-mediated hydrogen bonds (Figure 3C and Figure 4A). Moreover, Leu95 and His97 were conserved in other homologous pheromone-binding proteins (Figure S3), and these conservations may play a key role in pheromone binding (Figure 4).

In the apo-HarmPBP1, there was a channel through the ligand binding pocket (Figure S4A). However, one opening was covered in the HarmPBP1/*Z*9-16:Ald complex (Figure 1B and Figure S4B), because three C-terminal additional residues were solved and Leu161 occupied the opening. These three residues adopted a loop conformation that was greatly different from those in the complex of BmorPBP-bombykol and AtraPBP-*Z*11*Z*13-16:Ald [5,13]. Although the C-terminal residue Leu161 was somewhat away (5.6 Å) from the ligand, it had hydrophobic interactions with side-chains of Phe63, Phe60, and Ser36 and thus stabilized the hydrophobic binding pocket. The only one opening in the HarmPBP1/*Z*9-16:Ald complex was surrounded by helices α5, α6, and the α3α4 loop (Figure S4B). The *Z*9-16:Ald was in an elongated conformation, with one end entering the cavity through the opening formed by His122, Ala125 in α5, Lys138 in α6, and Lys94 and Leu95 in α3α4 loop (Figure 4B). In the apo-HarmPBP1 structure, part of the Lys138 side chain was flexible (Figure S4A). The binding of *Z*9-16:Ald induced conformational changes of Lys94 and Lys138 side chains and significantly hindered the access of the ligand to the solvent (Figure S4B). Thus, we hypothesize that the entry of ligand into the pocket is followed by a shift of Lys94 and Lys138, which act as a lid. The conformational rearrangements might trigger the lid to cover the opening of the pocket.

### 2.4. A New Releasing Mechanism at Low pH Conditions

Various studies [5,10,13] suggested significant pH-dependent conformational changes in lepidopteran PBPs. The C-terminus would form an additional helix α7 under low pH conditions, occupying the corresponding pheromone binding pocket. To know whether HarmPBP1 has a similar mechanism, we solved the structure of the apo-HarmPBP1 at acid conditions (Table 1). Surprisingly, the conformations of the apo-HarmPBP1 at pH 5.5 were very similar to those at pH 7.5 with an overall RMSD of 0.5 Å. Therefore, the above-mentioned significant structural changes influenced by pH may not exist in HarmPBP1. Moreover, no C-terminal additional α-helix was observed, and no residues were found in the cavity at pH 5.5. The cavity volume of the HarmPBP1 decreased from 266 Å^3^ to 211 Å^3^ when the pH changed from 7.5 to 5.5. The smaller cavity in HarmPBP1 would be not enough to accommodate an additional helix α7, which is found in other PBPs under low pH conditions [5,10,13].

**Figure 5:**
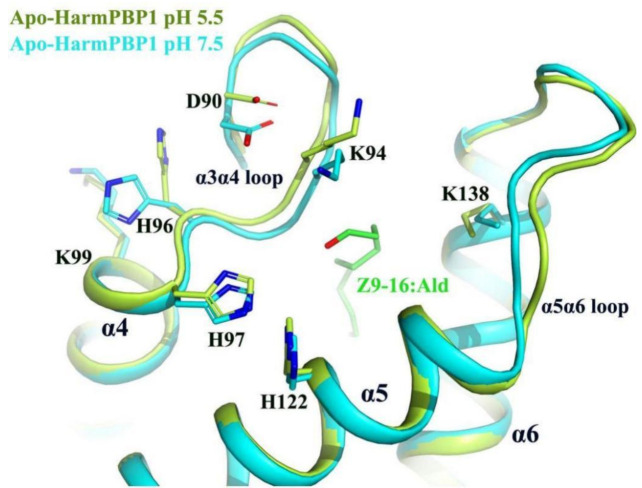
Superimposition of apo-HarmPBP1 at pH 5.5 (limon) on apo-HarmPBP1 at pH 7.5 (cyan). The side chain of His96 in the α3α4 loop changed greatly due to the protonation. The α3α4 loop and the α5α6 loop of the HarmPBP1 at pH 5.5 moved outward so that the pocket formed a larger opening. Z9-16:Ald (green) was docked from the HarmPBP1/Z9-16:Ald complex after superimposition.

Nevertheless, our structures uncovered a new releasing mechanism at low pH conditions. There were three residues (His96, His97, His122) that were strictly conserved across all known lepidopteran PBPs, suggesting that their role in the PBP function may be similar (Figure S3). A decrease of pH from 7.5 to 5.5 would result in the protonation of the imidazole rings of His96, His97, and His122 (Figure 5), which were in close contact, and therefore mutual repulsion probably would occur. Moreover, protonation changed the position of His96, which in turn increased its interaction with Asp90 located at the α3α4 loop and simultaneously increased its repulsive ability with Lys99. These changes triggered the movement of the α3α4 loop about 1.0 Å, enabling a larger opening of the pocket at pH 5.5 (Figure 5). In addition, the α5α6 loop around the opening also moved out about 1.5 Å. These movements might increase the interacting distance between the protein and the ligand. Therefore, the binding ability of HarmPBP1 to its ligand was weaker at low pH, and the fluorescence-binding experiment also proved this. The affinity of HarmPBP1 to *Z*9-16:Ald and *Z*11-16:Ald was measured under neutral (pH 7.5) and acidic (pH 5.5) conditions, respectively. HarmPBP1 showed a higher binding affinity to a nonspecific ligand 1-NPN at pH 7.5 (1.79 ± 0.14 μM) than at pH 5.5 (4.49 ± 0.41 μM) (Figure S5A). Further competitive binding assays showed that HarmPBP1 exhibited reduced binding activity for two principal pheromones *Z*11-16:Ald and *Z*9-16:Ald at low pH (Table 2, Figure S5). In summary, it is believed that the α3α4 loop, providing an entrance for the ligand, becomes destabilized upon protonation of one or all of three histidine residues at low pH.

## 3. Discussion

In this work, we solved three crystal structures of HarmPBP1 at two pH values and its complex with ligand *Z*-9-hexadecenal. Minor conformational changes occurred when the ligand bound to the pocket, during which C-terminal additional residues and the lid (Lys94 and Lys138), respectively, covered the two openings of the channel in the apo-HarmPBP1. Moreover, the two residues of the lid were not conserved among lepidopteran PBPs (Figure S3), revealing a unique mechanism. In addition, a new releasing mechanism at low pH conditions was found for HarmPBP1.

Our crystal structure of HarmPBP1 could further explain the results of former mutation experiments [16]. In that study, it was proven that four residues—Phe39, Phe63, Trp64, and Phe146—were the key residues involved in ligand recognition and interaction. Fluorescence assays revealed that all four mutants showed lower affinities to *Z*11-16:Ald [17] and *Z*9-16:Ald compared with the wild type. Mutants F39A and F146A exhibited strong reductions in affinities to both pheromone components *Z*9-16:Ald and *Z*11-16:Ald. Our complex structure revealed that the conjugated double bond of *Z*9-16:Ald was stuck in the middle of the aromatic rings of these two residues, further confirming their importance for substrate binding. The reduced affinities reported for the mutants might be explained by the disruption of the hydrophobic interactions between the protein and the ligand. Moreover, a HarmPBP1M mutant without the C-terminal nine residues had strong binding affinities to both ligands compared with the wild type at acidic condition, but not at neutral condition (Figure S6). The results indicate that the C-terminal nine residues of HarmPBP1 protein play an important role in the process of releasing ligands. Interestingly, a groove that formed between α1b and α2 only at pH 5.5 (Figure S4C–E) might have provided a nonspecific binding site for the C-terminal nine residues and thus would promote the release of ligands. Except for the strong hydrophobic interactions, a few hydrophilic interactions were also observed in the HarmPBP1/*Z*9-16:Ald complex structure. The aldehyde group of the ligand formed a strong hydrogen bond with the main chain of Leu95 and a weak water-mediated hydrogen bond with the side chain of His97 (Figure 3C and Figure 4A). Due to the weak selectivity of these hydrogen bonds, these results revealed that the binding specificity of the protein might not be very high. Consistently, *Z*11-16:Ald had a slightly weaker binding affinity to HarmPBP1 compared with *Z*9-16:Ald.

Previous research has indicated that the affinity of Lepidoptera PBPs is affected by pH change, facilitating the release of pheromones. Currently, two mechanisms have been reported. One mechanism is the internalization of the C terminus in form of a helix α7 into the binding cavity at acidic pH [5,9] (Figure S1A). Another mechanism is uncovering the ligand-binding cavity with the reorientation of helices α1, α3, and α4 but without forming the C-terminal helix α7 [8] (Figure S1B). In both mechanisms, the N-terminal helices (α1a for BmorPBP, α1a and α1b for ApolPBP) unfold and the overall conformation of the protein changes significantly. Here, we showed the third mechanism of pH-induced release of pheromones. The overall structure of HarmPBP1 at pH 5.5 was very similar to that at pH 7.5, with an RMSD of 0.5 Å. Moreover, the arrangements of both proteins in the crystal packing were different, revealing that the little structural difference was not caused by experimental errors. Subtle structural changes in the lid and the opening were observed. Acidic pH caused protonation of His96, His97, and His122 at the surface and provided the driving force to enlarge the opening of the ligand-binding cavity. The mechanism may be the most efficient and the best energy-saving method. Therefore, pheromone-binding and -releasing mechanisms were found to be different in the PBPs, consistent with the different physiological functions and structures of the ligands.

Insect PBPs have evolved significant structural differences that make them display different cavities and different mechanisms to bind and release diverse ligands. In this work, we found that HarmPBP1 adopts a new mechanism. Both apo-HarmPBP1 at different pH values and HarmPBP1/*Z*9-16:Ald were found to have similar conformation, suggesting that minor conformational rearrangements may also regulate ligand binding and releasing. We also demonstrated that the specific conformation of apo-HarmPBP1 was greatly different from other PBPs. Taken together, this study provides a structural basis for designing small inhibitors to control cotton bollworms, one of the agricultural pests that occur worldwide.

## 4. Materials and Methods

### 4.1. Expression and Purification of Recombinant HarmPBP1

The cDNA encoding residues 27-170 of HarmPBP1 (UniProtKB: F5ANH9) were cloned into the *Nco* I and *Xho* I restriction sites of the pET-32b modified vector, in which the thrombin site was replaced by a tobacco etch virus (TEV) protease cleavage site [18]. The cloned sequence was verified by sequencing and the correct plasmid was transformed into *E. coli* BL21 (DE3) cells. Cells were grown at 37 °C until OD_600_ reached 0.6-0.8, and protein expression was induced with 0.2 m*M* isopropyl β-D-thiogalactopyranoside (IPTG) at 18 °C for 12 h. Cells were harvested by centrifuging at 4000× *g* for 10 min and then resuspended in the lysis buffer containing 20 m*M* Tris-HCl (pH 8.0), 500 m*M* NaCl, 10 m*M* imidazole, 0.1% Triton X-100, and 1 m*M* PMSF (phenylmethanesulfonyl fluoride). After sonication, the cell lysates were centrifuged at 18,300× *g* for 30 min, and then the supernatant was filtered with 0.4 μm filter before being loaded onto a profinity^TM^ IMAC Ni-charged resin (Biorad, Cat. #156-0137) column equilibrated with lysis buffer and eluted with a 20–400 m*M* imidazole gradient. The N-terminal His-tag was removed by digestion with TEV protease containing a His-tag. After TEV protease digestion, the sample was passed over a second Ni-charged resin (Biorad) to remove the cleaved His-tag and TEV protease. Then, the proteins were loaded onto the Q sepharose^TM^ high-performance (GE Healthcare, Cat. #17-1014-03) column. Fractions containing the target protein were judged by SDS-PAGE analysis. The eluted protein was further purified by size-exclusion chromatography (Superdex^TM^ 200 10/300 GL, GE Healthcare, Cat. #17-5175-01) with buffer containing 20 m*M* Tris-HCl (pH 8.0), 150 mM NaCl, and 5 mM β-Me (β-Mercaptoethanol). Fractions containing the target protein were pooled and concentrated to 10 mg/mL for crystallization experiments.

### 4.2. Protein Crystallization and Data Collection

Initial crystallization trials were performed at 16 °C using the sitting-drop vapor-diffusion method. After two weeks, crystals could be observed in several conditions. After optimizations of crystallization conditions, large crystals were obtained in the buffer comprising 1.3 M Na-citrate, 0.1 M Tris-HCl (pH 7.5~8.5). The crystal structure of apo-HarmPBP1 at acid condition was obtained by soaking for 1 h under pH 5.5 conditions, similar to the references [19,20].

The HarmPBP1/ligand complex was prepared by adding ligands to the protein solution in a molar ratio of 10:1. After incubating overnight, the complex was concentrated to 25 mg/mL for crystallization trials. Crystallization trials were performed using the hanging-drop method by mixing 1 μL of the protein solution with 1 μL of well solution. It took several weeks to grow small crystals in the buffer comprising containing 1.5 M Na-citrate and 0.1 M Tris-HCl (pH 8.5) at 16 °C. After a series of optimizations of crystallization conditions including protein concentration, precipitants, pH, and salts, larger crystals with better diffraction were obtained in the buffer comprising 1.3 M Na-citrate and 0.1 M Tris-HCl (pH 8.5).

Crystals were frozen for data collection in the above crystallization buffer containing 25% glycerol. Data were collected on beamline BL17U1 at the Shanghai Synchrotron Radiation Facility. Data were indexed, integrated, and scaled using HKL2000 [21]. Data collection and processing statistics are shown in Table 1.

### 4.3. Structure Determination and Refinement

Apo-HarmPBP1 structure (pH 7.5) was solved by the molecular replacement program BALBES [22] using the BmorPBP structure (pdb: 2FJY) [9] as a model. We removed unstructured loops and deleted disordered side chains in the density map, and the initial model was refined by the program REFMAC5 [23]. The additional residues were rebuilt manually by COOT [24], and the model was further refined by using REFMAC5. After 30 more cycles of manual rebuilding by COOT and refinement with REFMAC5 [21] by calculating hydrogens and anisotropic refinement, the structure was refined to 1.3 Å with an *R_work_* of 17.9% and an *R_free_* of 19.8% (Table 1). Structures of apo-HarmPBP1 at pH 5.5 and HarmPBP1/*Z*9-16:Ald complex were solved by molecular replacement program BALBES [22] using the apo-HarmPBP1 (pH 7.5) as a model. Structure of apo-HarmPBP1 at pH 7.5 (PDB ID: 7VW8), structure of apo-HarmPBP1 at pH 5.5 (PDB ID: 7VW9), and structure of HarmPBP1/*Z*9-16:Ald complex (PDB ID: 7VWA) have been deposited in the RCSB Protein Data Bank (http://www.rcsb.org/pdb (accessed on 30 December 2021)). The cavity volume mentioned above was calculated by the program VOIDOO [25].

### 4.4. Fluorescence Binding Assays

Fluorescence binding assays were conducted on an F-380 fluorescence spectrophotometer (Tianjin Gangdong Sci. & Tech.) to further investigate the binding characteristics of HarmPBP1. N-phenyl-1-naph-thylamine (1-NPN), Z11-16:Ald, and Z9-16:Ald (>98%) were purchased from Sigma. The principal pheromone components of *H. armigera*, *Z*11-16:Ald, and *Z*9-16:Ald were used as the competitors, and 1-NPN was used as the fluorescent ligand in a 1 cm light path quartz cuvette. Both the emission and excitation slit widths were 10 nm. Fluorescence of 1-NPN was excited at 337 nm, and the emission spectra were recorded between 390 and 490 nm. Fluorescence measurements were performed according to the reference [16]. Solutions of 2 μM proteins in the corresponding buffer and 2 μM 1-NPN were titrated with 1 mM solutions of each ligand in methanol to a final concentration of 4 μM (pH 7.5) and 10 μM (pH 5.5). Dissociation constants of the competitors were calculated from the corresponding IC_50_ values (concentrations of ligands halving the initial fluorescence value of 1-NPN) using the equation: K_i_ = [IC_50_]/(1 + [1-NPN]/K_1-NPN_); [1-NPN] was the concentration of free 1-NPN, and K_1-NPN_ was the dissociation constant of the complex protein/1-NPN, which was calculated from the binding curve using Prism 5.0 (GraphPad Software Inc.). Note that the titration may be terminated when the fluorescence intensity is less than IC_50_ because only the IC_50_ value is needed in the calculation.

## Figures and Tables

**Table 1:** Data collection and refinement statistics of HarmPBP1 structures.

	Apo-HarmPBP1 at pH 7.5 (PDB ID:7VW8)	Apo-HarmPBP1 at pH 5.5 (PDB ID:7VW9)	HarmPBP1/*Z*9-16:Ald Complex at pH 8.5 (PDB ID: 7VWA)
Wavelength, Å	0.9792	0.9792	1.0000
Space group	*P*2_1_	*P*2_1_	*P*2_1_
Cell dimensions			
*a*, *b*, *c*, Å	32.03, 32.60, 54.79	32.43, 33.38, 55.38	32.91, 33.36, 56.04
*α*, *β*, *γ*, °	90, 97.88, 90	90, 99.66, 90	90, 98.79, 90
Resolution, Å	50-1.30 (1.32–1.30) ^a^	50-2.05 (2.09–2.05) ^a^	50-2.09 (2.13–2.09) ^a^
*R*merge, %	6.6 (59.9)	5.0 (21.4)	5.7 (30.2)
*I*/σ*I*	17.7 (1.8)	41.2 (7.6)	31.1 (3.0)
Completeness, %	93.1 (97.5)	95.6 (93.5)	98.2 (81.0)
Redundancy	4.3 (3.9)	3.5 (3.1)	4.9 (3.7)
Refinement			
Resolution, Å	50-1.3 (1.33–1.30)	50-2.05 (2.10–2.05)	50-2.10 (2.15–2.10)
No. of unique reflections	21224 (736)	6834 (465)	6682 (432)
*R*_work_/*R*_free_, %	17.9/19.8 (22.3/24.9)	22.2/26.5 (28.4/36.7)	19.9/23.6 (24.2/37.1)
No. of atoms (protein/ligand/water)	1048/0/62	1015/0/56	1059/17/40
Average B factor (Å^2^) (protein/ligand/water)	21.44/0/30.79	43.69/0/47.31	31.07/37.11/36.18
rms deviations			
Bond lengths, Å	0.008	0.007	0.009
Bond angles, °	1.378	0.994	1.515
Ramachandran Plot, % ^b^	95.9/4.1/0/0	91.1/8.9/0/0	92.9/7.1/0/0

^a^ Statistics for highest resolution shell. ^b^ Percent of residues in most favored, additional allowed, generously allowed, and disallowed regions of the Ramachandran plot.

**Table 2:** Binding affinities (μM) of two major sex pheromones to HarmPBP1 at pH 7.5 and pH 5.5.

Ligand	pH 7.5	pH 5.5
(*Z*)-11-Hexadecenal	0.67 ± 0.05	13.09 ± 1.78
(*Z*)-9-Hexadecenal	0.56 ± 0.05	13.61 ± 1.33

Data represent the mean values ± S.E.M of three independent replicates.

## Data Availability

Final refined coordinates and structure factors have been deposited in the Protein Data Bank (PDB) under accession codes 7VW8, 7VW9, and 7VWA.

## Supplementary Materials

The following are available online at https://www.mdpi.com/article/10.3390/ijms23031190/s1.
