# Supplementary material for: Structural Insights into the Ligand-Binding and -Releasing Mechanism of Helicoverpa armigera Pheromone-Binding Protein PBP1
Source: Int J Mol Sci. 2022 Jan 21;23(3):1190. doi: 10.3390/ijms23031190 (PMC8835341; doi:10.3390/ijms23031190)
Supplement: Supplementary file 1 [file ijms-23-01190-s001.zip › ijms-1560317-supplementary.pdf]

Supplementary Data

Fig. S1. Two mechanisms of the pH-induced conformational transition.

Fig. S2. The purify and structural comparison of HarmPBP1.

Fig. S3. Structure-based sequence alignment of HarmPBP1 with BmorPBP, AtraPBP1, and ApolPBP1.

Fig. S4. **The channel in apo-HarmPBP1 at pH 7.5 (A) and the opening in HarmPBP1/Z9-16:Ald complex (B)**.

Fig. S5. **Competitive binding curves of *H. armigera* main sex pheromone components to HarmPBP1 at different pHs.**

Fig. S6. Competitive binding curves of *H. armigera* main sex pheromone components to HarmPBP1M mutant at different pHs.

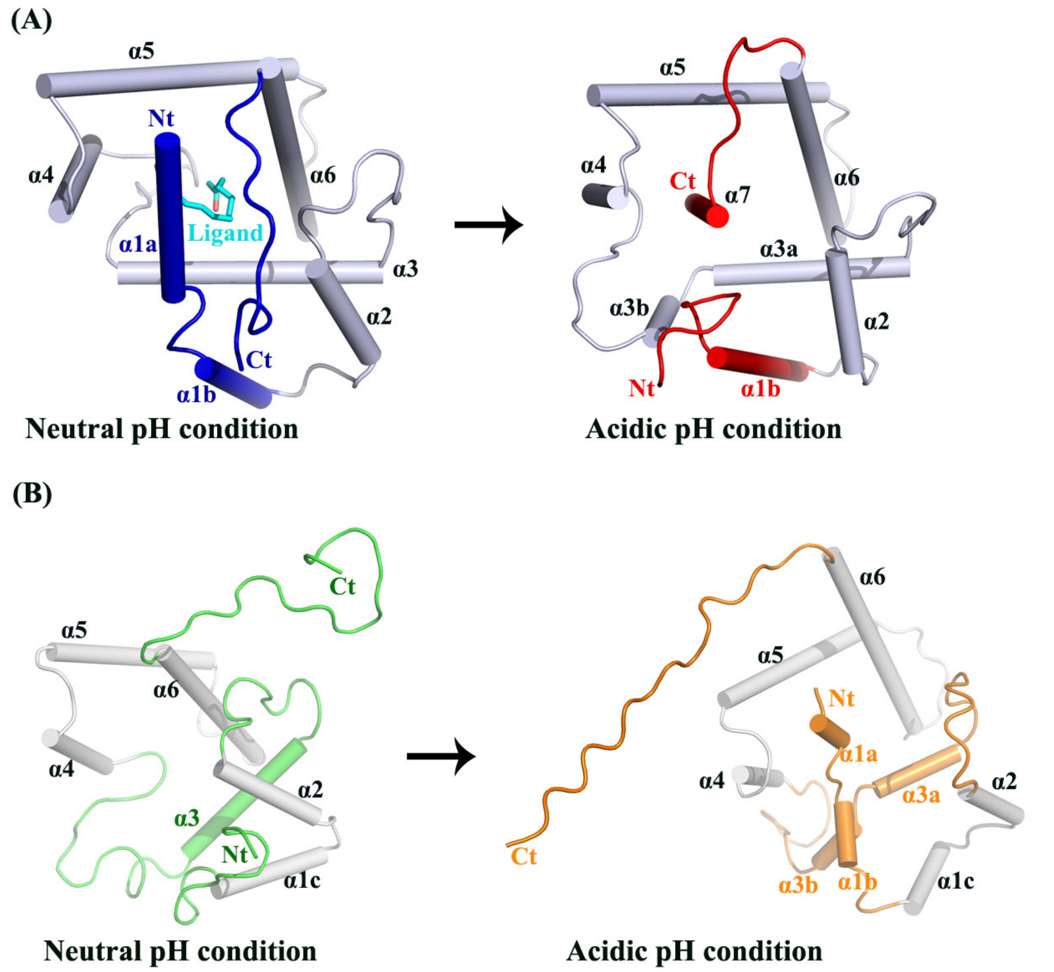

Figure S1. Two mechanisms of the pH-induced conformational transition. (A) The C terminus of the acidic BmorPBP structure formed an additional α-helix in the protein core, occupying the corresponding pheromone-binding site, and extruding ligands (cyan) [1,2]. Helical fragments are shown as cylinders, colored blue in the neutral pH structures and red in the acidic pH. (B) A reorientation of helices α1 and α3 at acidic pH caused by protonation of histidines in ApolPBP provides the driving force of pheromone-releasing [3]. Helical fragments are shown as cylinders, colored green in the neutral pH and orange in the acidic pH structures. Light grey cylinders show the rest of the structure.

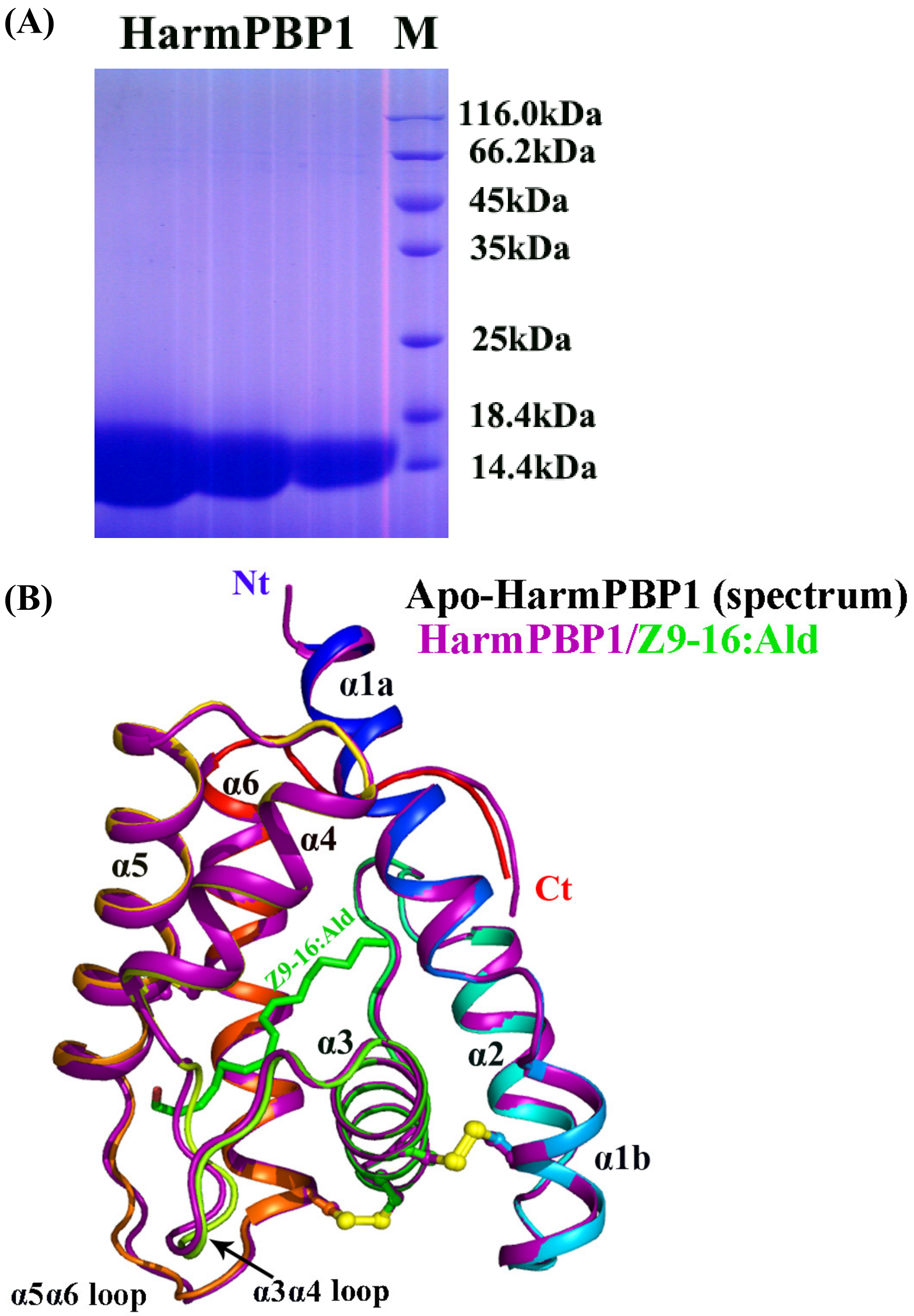

Figure S2. The purify and structural comparison of HarmPBP1. (A) The SDS-PAGE of the purified apo-HarmPBP1. The apo-HarmPBP1 protein was purified by size-exclusion chromatography, and then the collected fractions were analyzed by SDS-PAGE. (B) Comparison of apo-HarmPBP1 (color: spectrum) and Z9-16:Ald-bound (magenta) of HarmPBP1 reveals conformational changes in α3α4 loop and α5α6 loop. Z9-16:Ald, green.

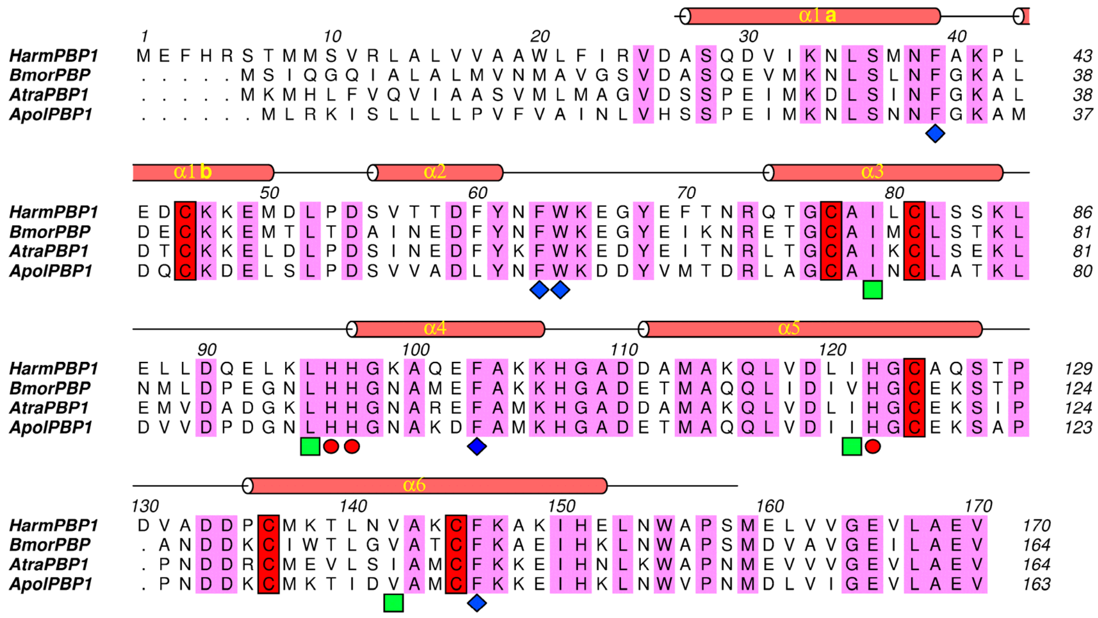

Figure S3. Structure-based sequence alignment of HarmPBP1 with BmorPBP, AtraPBP1, and ApolPBP1. The figure was made by Align [4]. The secondary structures of apo-HarmPBP1 are shown above the sequence, composed of six helices. The six conserved cysteine residues were boxed in red. The corresponding residues involved in hydrophobic contacts with the pheromones were marked with solid green squares and solid blue diamonds (five conserved aromatic residues). Residues forming histidine protonation switch were marked with solid red cycles. The conserved residues are shown in pink.

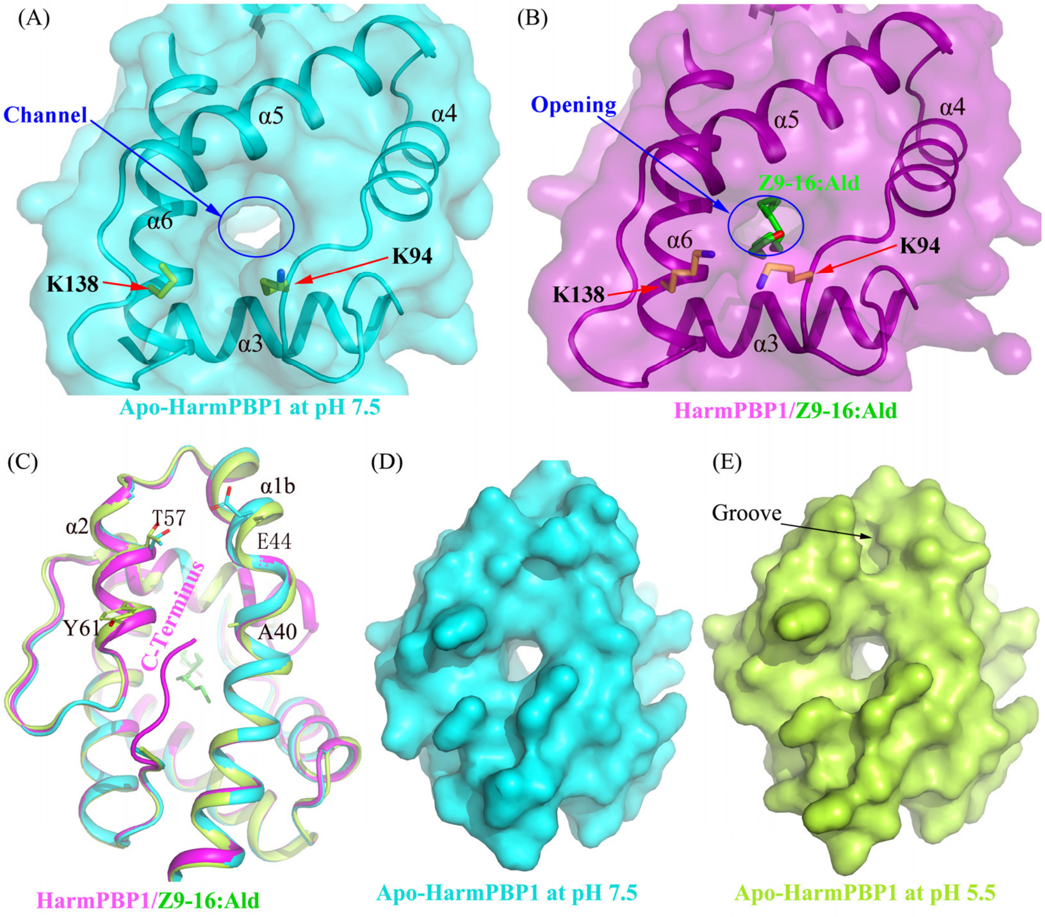

Figure S4. **Surfaces of HarmPBP1.** The channel in apo-HarmPBP1 at pH 7.5 (A) and the opening in HarmPBP1/Z9-16:Ald complex (B). The opening is composed of α5, α6, and the α3α4 loop. Interactions of Z9-16:Ald with K94 and K138 induce conformational changes of side chains in K94 and K138 compared with those in the apo structure. The HarmPBP1/Z9-16:Ald complex (B) is shown in magenta, and the corresponding region of the apo-HarmPBP1 (A) structure is shown in cyan. The Z9-16:Ald is shown in green. K94 and K138 are shown in sticks. (C) Comparison of three structures around the C-terminus. Surfaces of apo-HarmPBP1 at pH 7.5 (D) and apo-HarmPBP1 at pH 5.5 (E). Note that a groove formed between α1b and α2 is found only at pH 5.5.

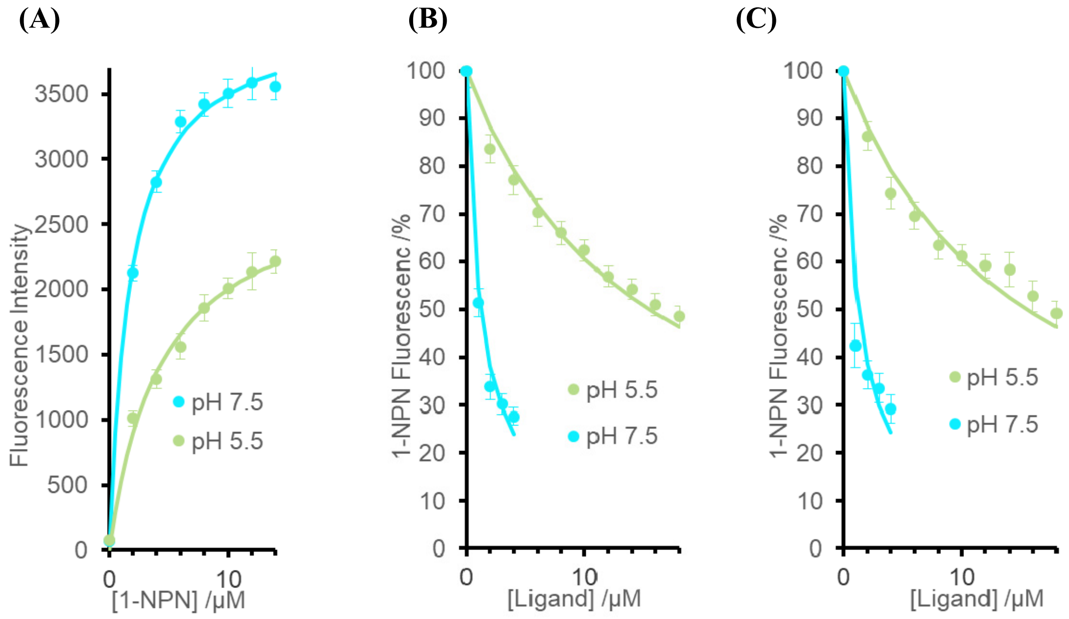

Figure S5. **Competitive binding curves of *H. armigera* main sex pheromone components to HarmPBP1 at different pHs. (A)** Binding curves of 1-NPN binding to HarmPBP1 at pH 7.5 and pH 5.5. Competitive binding curves of Z11-16:Ald **(B)** and Z9-16:Ald **(C)** to HarmPBP1 in the presence of 1-NPN. pH 5.5, limon; pH 7.5, cyan. The experiments were repeated three times.

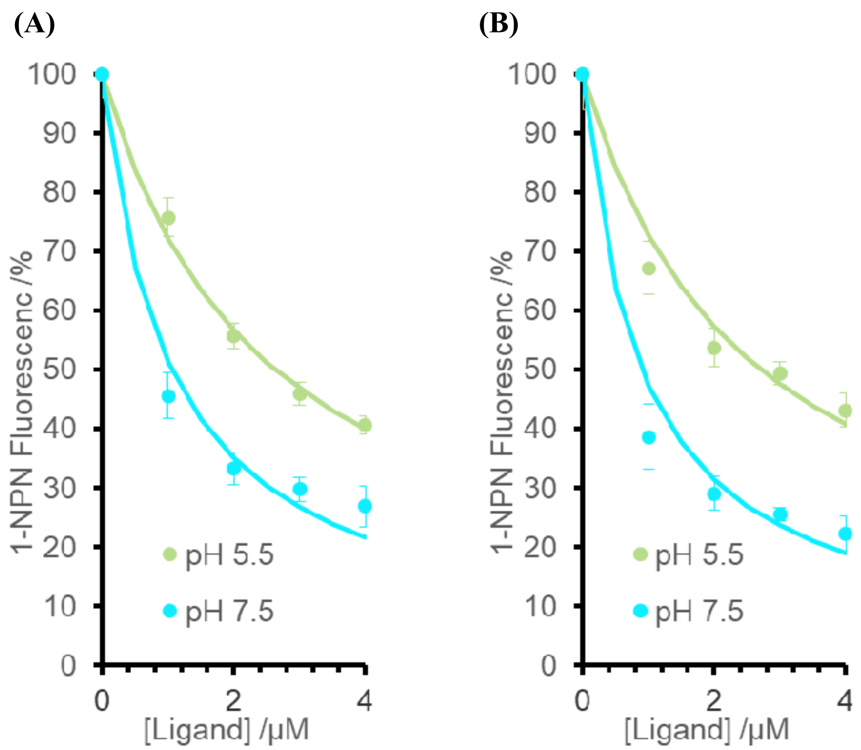

Figure S6. Competitive binding curves of *H. armigera* main sex pheromone components to HarmPBP1M mutant at different pHs. Competitive binding curves of Z11-16:Ald (A) and Z9-16:Ald (B) to HarmPBP1 in the presence of 1-NPN. pH 5.5, limon; pH 7.5, cyan. The experiments were repeated three times. Note that the HarmPBP1M mutant deletes C-terminal nine residues. At pH 7.4, the binding affinities of Z11-16:Ald and Z9-16:Ald to HarmPBP1M are 0.56±0.033 µM and 0.50±0.027 µM, respectively. At pH 5.5, the binding affinities of Z11-16:Ald and Z9-16:Ald to HarmPBP1M are 1.79±0.27 µM and 1.99±0.11 µM, respectively.

Supplementary references:

1. Sandler, B.H.; Nikonova, L.; Leal, W.S.; Clardy, J. Sexual attraction in the silkworm moth: structure of the pheromone-binding-protein-bombykol complex. *Chem Biol* **2000**, *7*, 143-151, doi:10.1016/s1074-5521(00)00078-8.
2. Lautenschlager, C.; Leal, W.S.; Clardy, J. Coil-to-helix transition and ligand release of Bombyx mori pheromone-binding protein. *Biochem Biophys Res Commun* **2005**, *335*, 1044-1050, doi:10.1016/j.bbrc.2005.07.176.
3. Zubkov, S.; Gronenborn, A.M.; Byeon, I.J.; Mohanty, S. Structural consequences of the pH-induced conformational switch in A.polyphemus pheromone-binding protein: mechanisms of ligand release. *J Mol Biol* **2005**, *354*, 1081-1090, doi:10.1016/j.jmb.2005.10.015.
